# Computational methods and resources for the interpretation of genomic variants in cancer

**DOI:** 10.1186/1471-2164-16-S8-S7

**Published:** 2015-06-18

**Authors:** Rui Tian, Malay K Basu, Emidio Capriotti

**Affiliations:** 1 Division of Informatics, Department of Pathology, University of Alabama at Birmingham, 619 19th St. South, 35249 Birmingham (AL), USA; 2 Department of Clinical and Diagnostic Sciences, University of Alabama at Birmingham, 619 19th St. South, 35249 Birmingham (AL), USA; 3 Department of Biomedical Engineering, University of Alabama at Birmingham, 619 19th St. South, 35249 Birmingham (AL), USA

## Abstract

The recent improvement of the high-throughput sequencing technologies is having a strong impact on the detection of genetic variations associated with cancer. Several institutions worldwide have been sequencing the whole exomes and or genomes of cancer patients in the thousands, thereby providing an invaluable collection of new somatic mutations in different cancer types. These initiatives promoted the development of methods and tools for the analysis of cancer genomes that are aimed at studying the relationship between genotype and phenotype in cancer.

In this article we review the online resources and computational tools for the analysis of cancer genome. First, we describe the available repositories of cancer genome data. Next, we provide an overview of the methods for the detection of genetic variation and computational tools for the prioritization of cancer related genes and causative somatic variations. Finally, we discuss the future perspectives in cancer genomics focusing on the impact of computational methods and quantitative approaches for defining personalized strategies to improve the diagnosis and treatment of cancer.

## Background

The advances in high-throughput sequencing techniques are allowing us to identify a large number of genetic variants in human [1,2] and understand the relationship between genotype and phenotype in many genetic disorders [3]. In contrast to Mendelian disorders, in which a disease is the result of inherited variations present in a single gene or a small set of genes, cancer is mainly driven by accumulated somatic variations in multiple genes. These mutations enable a particular subpopulation of cells to proliferate and survive more efficiently than their neighbors [4-6]. The different types of somatic genetic variations detected in cancer samples vary from single nucleotide variants, short insertion and deletion (indels), large copy number alterations, to structural rearrangements [7]. Thus, the identification of causative genomic variations is key point for understanding the mechanism of cancer. The solution of this challenging task is limited by the accuracy of sequencing technology and the large number of genetic alterations observed in cancer genome. Although current whole-exome sequencing is performed with a coverage between 100x to 150x, still many false positive arise from sequencing error, inaccurate alignments and admixture of noncancer and different subclonal cells [8].

Even with a perfect sequencing approach the detection of causative variants remains a complex task. Indeed, among somatic variants, a large fraction, defined as "passengers", have no impact on the neoplastic process. But a smaller set of genetic variants, referred to as "drivers", provide a selective growth advantage, estimated to be minute (~0.4%), to the cell [9]. The accumulation of those variants and their effect, over the years, can generate a tumor. According to a recent review [9], somatic variations observed in common solid tumors affect on average between 33 and 66 genes. The predominant type of variations is the single nucleotide substitution, which accounts for more than 90% of somatic changes [1]. The role of these genes in tumorigenesis is determined by the functional impact of somatic variants they harbor. In general, mutations that enhance the function of oncogenes and impair the activity of tumor suppressor genes result in a selective growth advantage for the cancer cell.

**Figure 1:**
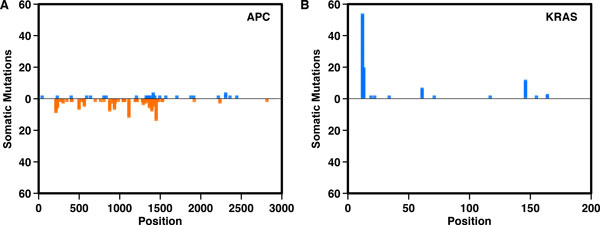
**Number of missense and stop gain somatic mutations in APC and KRAS detected in 220 samples of colon adenocarcinoma from TCGA studies**. In blue and orange are reported the nonsynonymous and stop gain somatic variants respectively. For APC we detected a total number of 265 somatic variants 28 of which are nonsynonymous and 150 stop gain. Among the 28 stop-gain variants only 6 are recurrent. For KRAS we detected a total number of 101 somatic variants 97 of which are nonsynonymous. Among the observed nonsynonymous variants 53 are observed in position 12 and only 6 are not recurring.

The frequency of observed somatic variants draw a mutational landscape made up of few "mountains" and a large number of "hills" that respectively correspond to frequently and rarely mutated genes across tumor samples [9]. Among the frequently mutated genes, the distribution of somatic variants can reflect the differences between oncogenes and tumor suppressor genes. Oncogenes tend to be recurrently mutated at the same amino acid positions, whereas tumor suppressor genes are generally mutated in various positions throughout their length. An example is provided in Figure 1, which shows the frequencies of somatic mutation in APC (a tumor suppressor gene) and KRAS (an oncogene) from 220 samples of colon adenocarcinoma from The Cancer Genome Atlas (TCGA) consortium. In this example, ~60% of the variants in APC are stop-gain which cause loss of function. In contrast, ~50% of the mutations in KRAS are missense variants in position 12 which are expected to increase the protein function.

Given that the cancer is a result of interplay of various types of genetic changes, understanding of the role of somatic mutations in tumorigenesis is a complex problem in which different combinations of somatic variants affect different network of genes and associated pathways. Nevertheless, significant breakthroughs have been made in the development of computational methodologies which allow us to identify new driver mutations and genes by analyzing large sets of patients with different tumors [10]. Currently, the main bioinformatics challenges in the analysis of cancer genome consist of the following:

1. Robust pipelines for the detection of genetic variations;

2. Creation of a benchmark dataset of cancer driver mutations and genes;

3. Accurate methods for prioritizing cancer driver mutations and genes;

4. New algorithm for predicting the impact of somatic variants at pathway/ network level;

5. Translational approaches that make the results of computational analysis clinically applicable [11,12].

In this review we focus on the description of computational methods for cancer genome interpretation. First, we describe the basic steps for the detection of genetic variants. We summarize the currently available data sources for implementing and benchmarking new computational tools for cancer genome analysis. Second, we review the available methods for the prioritization of driver mutations and genes. We also include a section describing tools for predicting the impact of genetic variants at network level and methods for estimating the consistence of subclonal populations. Finally, we discuss the future perspective in the field, highlighting the contribution of computational approaches to cancer genomics. In contrast to the recently published cancer genomic reviews [11,13,14], we present an analysis of the somatic mutations in cancer and the data available online and provide a brief description and availability of selected computational tools for the analysis of cancer genomes. This review is targeted towards readers with background in computational biology and bioinformatics, who want to have quick introduction to the available resources and tools for the analysis of cancer genome.

### Variant calling, filtering and annotation

Accurate variant calling is the prerequisite of any cancer genome analysis, but it is hindered by several limitations. The first limitation stems from the inherent noise and errors in the sequencing technology. Errors are also introduced in the procedures of short reads alignment, especially in the low complexity regions of the genome. In addition, a recently published work showed that no single variant calling approach is able to comprehensively capture all genetic variations [15]. Thus, there is still room for improvement in variant calling algorithms.

In general, the variant calling procedure consist of 3 main steps:

1. Short read alignment and mapping to the reference genome.

2. Removing PCR duplicates, realignment and recalibration.

3. Variant calling, filtering and annotation.

**Figure 2:**
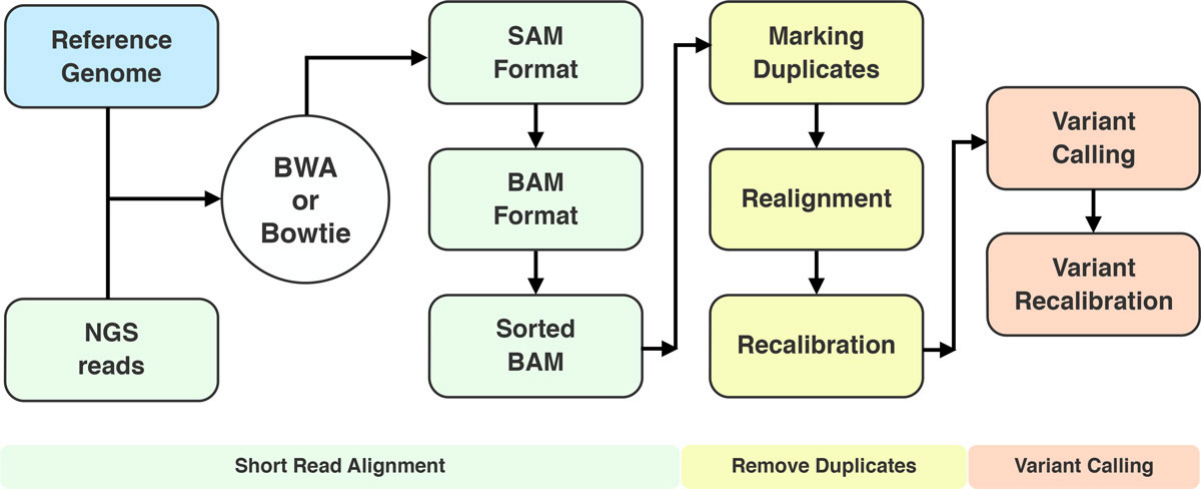
**A typical variant calling workflow**.

This procedure includes the calibration of pre- and post-experimental factors to identify reliable variants from the raw data. Such factors include sequencing coverage, single end or paired end sequencing, short read alignment, PCR duplicates, matched sample sequencing, variant calling algorithm, etc. [11,15,16]. A representation a typical variant calling pipeline is provided in Figure 2.

A plethora of tools have been developed for variant calling purposes. In cancer studies, a standard workflow for variant calling starts with the alignment against the reference genome using BWA [17] or Bowtie [18]. The resulting alignment (SAM file) is generally converted in binary format (BAM) and indexed using SAMtools [19]. The removal of PCR duplicates can be performed using Picard (http://sourceforge.net/projects/picard/). This step is followed by realignment and recalibration with GATK [20]. Finally, variant calling is performed using a standard variant caller like GATK or specialized tools such as VarScan 2 [21] and MuTect [22]. This step can be improved by the score recalibration that reduces the number of false positive calls. The final output of this pipeline is a VCF (variant calling format) file.

Although, theoretically the variant calling pipelines are straightforward, the results from different variant callers agree only on a small subset of variants. In a systematic test of several variant callers, namely GRISP [23], GATK, SAMtools, SNVer [24], VarScan 2, only ~50% of the SNPs are shared by all these tools, while the overlap of identified indels is even lower [15]. Recently, a similar study [16] has been performed by comparing the performances of another set of six variant callers, including EBCall [25], JointSNVMix [26], MuTect, SomaticSniper [27], Streika [28] and VarScan 2 [16]. Using experimentally validated SNVs as benchmark, it was reported that VarScan 2 and MuTect are among the best variant callers for analyzing matched normal and tumor samples.

After the variant calling file (VCF) has been obtained, a filtering procedure is often necessary for the downstream analysis. This filtering procedure aims to reduce the number of false positives corresponding either to low quality and common variants. In particular the common variants, which are assumed to have no implications in tumor development and progression, are filtered out by comparison with the germline polymorphisms collected in publicly available databases, such as dbSNP [29] or EVS (Exome Variant Server, http://evs.gs.washington.edu/EVS/). The most recent versions of dbSNP (build 142) contains more than 110 million human SNPs, while the current EVS data release (ESP6500SI-V2) include all the exome variant data from 6503 human samples. Some of the common tools used for filtering variants are SnpSift [30], GATK [20], VCFtools (http://vcftools.sourceforge.net).

Finally, variants are annotated by mapping each variant to their corresponding gene. This procedure is essential for understanding their functional consequences. Among the most popular tools for variant annotation are ANNOVAR [31], snpEff [32] and VEP [33].

### Cancer variation data and databases

Large-scale cancer genomic experiments, funded by several national and international consortiums, are generating an amount of data in the magnitude of PetaBytes (PB). The space needed to host the data only from The Cancer Genome Atlas (TCGA) is ~1.1PB (see https://cghub.ucsc.edu/summary_stats.html). The analysis of this data enabled the development of several meta databases and resources for the annotation of cancer genomes. In this section, we describe some of the repositories and databases available that collect somatic variants and driver genes putatively involved in the cancer.

#### Cancer mutation data

The Cancer Genome Atlas (TCGA) consortium, which began in United States in 2006, is a comprehensive and coordinated effort to understand the molecular basis of cancer using several genomic analysis techniques. The data generated by the experiments are made available through the TCGA Data Portal and the Cancer Genomics Hub (CGHub) [34]. After signing the certification data agreement it is possible to access data about 36 cancer types. The files containing the binary version of the short DNA sequence read alignments (referred as BAM files) can be downloaded using the GeneTorrent application available at the CGHub website (https://cghub.ucsc.edu/).

Since the downloading of all the BAM files remains costly in term of time and storage, it is advisable to get all the pre-processed variant files (VCFs) generated by different institutions within the TCGA consortium. Unfortunately, so far the organization of the data in the TCGA repository is not optimized and available data have to be manually selected. In particular, variant data from different platforms, ranging from SNP arrays to SOLID and Illumina sequencers, are reported in different file formats. Out of these files, the most informative ones are the VCF files, which contains the genetic variants for both normal and tumor samples. Currently, VCF file are provided only for a subset of the tumor types (~67%).

The International Cancer Genome Consortium (ICGC) was launched in 2007 to coordinate the efforts of characterizing more than 50 different cancer types from 25,000 patient genomes [35-37]. The results have been published and made publicly available [38-44]. To provide an overview of the variation data, we re-analyzed the somatic mutations publicly available at the ICGC portal, whereas the access to the germline variants requires the approval of a data agreement. The statistics of the release 17 (September 2014) of the ICGC data portal (https://dcc.icgc.org) show that in total 12,232 cancer genomes have been sequenced. In the samples from these donors, collected from 18 cancer primary sites, more than 9.8 million simple somatic mutations have been identified. A summary of the mutations detected for each cancer type is provided in Table 1. In this work, we consider samples from 42 sequencing projects corresponding to 33 cancer types. From the available data we excluded the samples from Acute Lymphoblast Leukemia for which only 3 mutations were detected. A brief description of the datasets analyzed in this manuscript as well as the final list of codes of each cancer project are provided in Section 1 of the Additional file 1 and Supplementary Table 1. In the data collected by the ICGC, breast cancer is the most studied cancer type in which more than 1,100 individuals have been screened.

**Table 1:** 

Cancer Type	Donors	Total Unique Mutations	Total Recurrent Mutations	Donors with Recurrent Mutations
BLCA	233	53,638	737 (1.4%)	219 (94.0%)
BOCA	66	1,422	7 (0.5%)	39 (59.1%)
BRCA	1,071	275,612	1,252 (0.5%)	929 (86.7%)
CLLE	109	5,292	4 (0.1%)	10 (9.2%)
CMDI	129	86	13 (15.1%)	113 (87.6%)
COAD	216	105,786	3,896 (3.7%)	215 (99.5%)
EOPC	11	25,575	2 (0.0%)	4 (36.4%)
ESAD	95	1,780,883	25,425 (1.4%)	95 (100.0%)
ESCA	88	7,256	17 (0.2%)	37 (42.0%)
GACA	9	1,014	0 (0.0%)	0 (0.0%)
GBM	268	19,852	324 (1.6%)	260 (97.0%)
KIRC	404	26,371	688 (2.6%)	372 (92.1%)
KIRP	156	12,932	218 (1.7%)	144 (92.3%)
LAML	75	60,203	7,623 (12.7%)	71 (94.7%)
LGG	279	13,083	432 (3.3%)	278 (99.6%)
LIAD	30	917	11 (1.2%)	19 (63.3%)
LICA	29	747,334	27,107 (3.6%)	6 (20.7%)
LINC	244	437,403	6003 (1.4%)	244 (100.0%)
LIRI	208	2,124,689	4161 (0.2%)	208 (100.0%)
LUSC	289	125,351	1400 (1.1%)	268 (92.7%)
MALY	44	311,297	686 (0.2%)	44 (100.0%)
NBL	41	137	2 (1.5%)	4 (9.8%)
ORCA	50	5,604	35 (0.6%)	38 (76.0%)
OV	181	919,769	869 (0.1%)	119 (65.7%)
PACA	504	1,630,944	6,098 (0.4%)	500 (96.5%)
PAEN	35	112,823	804 (0.7%)	32 (91.4%)
PBCA	248	130,608	1,231 (0.9%)	89 (35.9%)
PRAD	264	90,599	776 (0.9%)	256 (96.7%)
READ	80	23,499	556 (2.4%)	80 (100.0%)
RECA	105	475,986	6,067 (1.3%)	95 (90.5%)
SKCM	323	226,850	11,908 (5.2%)	323 (100.0%)
STAD	289	142,496	3,441 (2.4%)	276 (95.5%)
THCA	411	51,759	4,619 (8.9%)	311 (75.7%)
PanCancer	6,584	9,871,474	171,314 (1.7%)	6,296 (95.6%)

Somatic mutations in 33 tumor types from 42 projects from the ICGC release 17 (September 2014). *Donors*: total number of individual for each tumor type. *Total Unique Mutations*: number of somatic mutations in the whole genome for each cancer type removing repetitions. *Total Recurrent Mutations*: fraction of *Total Unique Mutations *observed at least in two donors for the same tumor type. *Donors with Recurrent Mutations*: fraction of donors with at least one recurrent somatic mutation.

To study the occurrence of somatic mutations across different donors, we performed a recurrence analysis calculating the *Fraction of Somatic Mutations *and the *Fraction of Donors *corresponding to different subsets of somatic mutations (Section 2 in Additional file 1).

**Figure 3:**
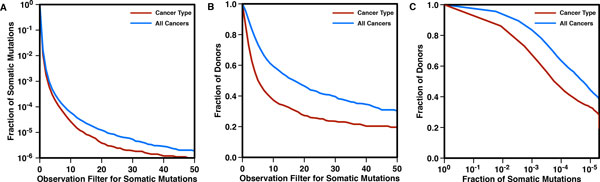
**Analysis of recurrent somatic mutations by cancer types (red) and all cancers together (blue)**. Plots A and B represent the complementary cumulative distributions (CCD) of the fraction of somatic mutations and affected donors respectively. They are calculated using an increasing level of mutation recurrence, which is defined as the number of times a somatic mutation is observed in different donors. In Panel C, is plotted the CCD of the fraction of affected donors as a function of the fraction of somatic variants. The curve is obtained considering an increasing threshold of mutation recurrence (Section 2 in Additional file 1).

The analysis of whole set of somatic mutations (PanCancer) revealed that a large fraction of them are occurring in a single patient and only ~1.7% are recurring more than once (see Table 1). This percentage decreases to 1.2% if the cancer types are considered separately. The plot in Figure 3A shows the *Fraction of Somatic Mutations *at different levels of *Mutation Recurrence*.

In addition, we calculated the *Fraction of Donors *covered by subsets of recurrent mutations (Figure 3B). Considering only the recurrent mutations, (observed at least in two donors), they are held by 96% of the individuals in all cancer types (Table 1). This percentage decreases to 82% when the cancer types are considered separately. In Figure 3C we plot the *Fraction of Somatic Mutations *and the *Fraction of Donors *of affected at different *Mutation Recurrence *thresholds. This curve allows us to estimate the fraction of donors affected by a subset of recurrent mutations that are more likely to have a functional impact. Although it is well accepted that each cancer sample is different and only a small fraction of variants are recurrent, we show that with mutations recurring more than 30 times (53 variants) explains a comparatively large fraction of patients (~40%). However, this data could be biased toward cancer types with higher number of samples and with higher of mutations detected.

**Figure 4:**
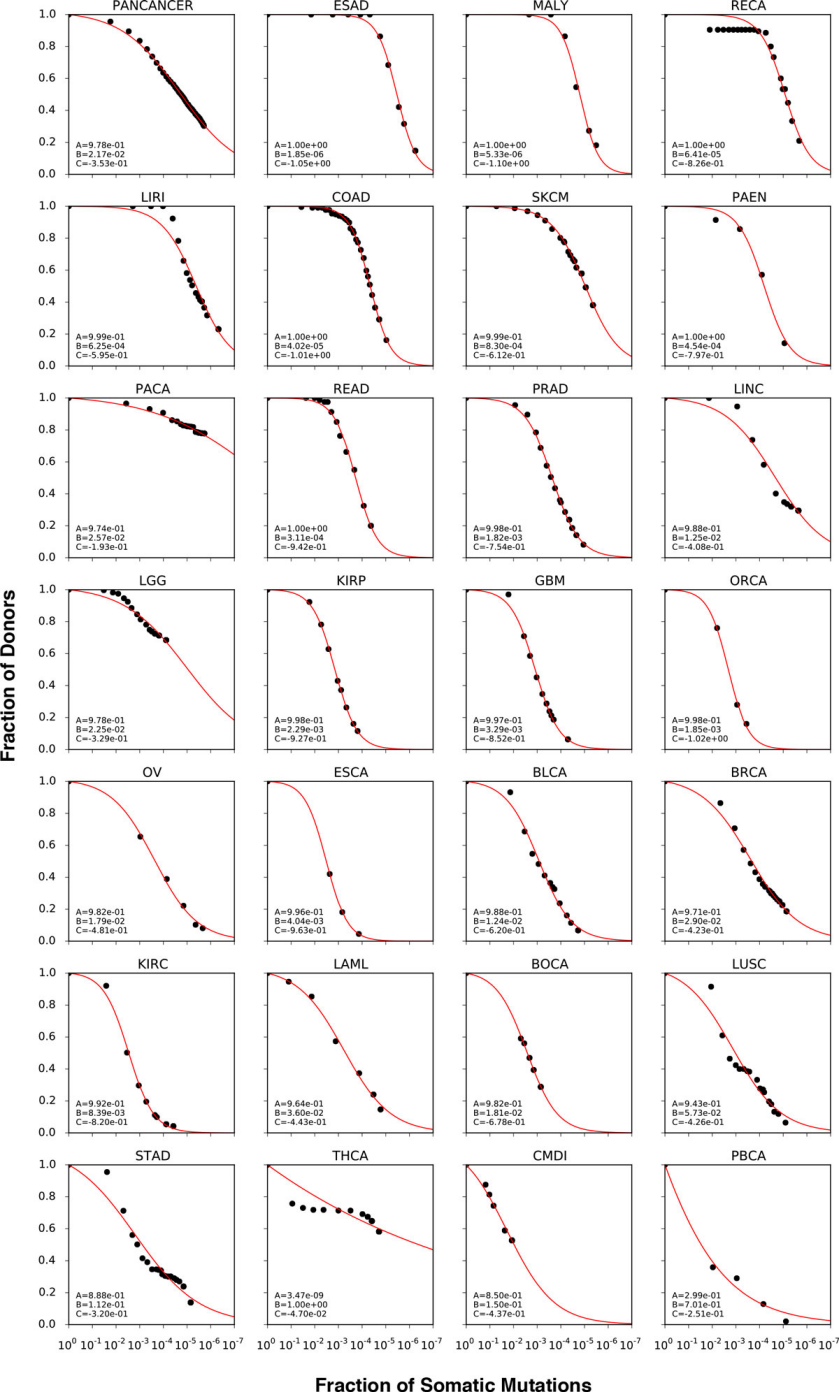
**Cancer-specific complementary cumulative distributions**. The plots shows the decreasing trend of the *Fraction of Donors *(Y axis) as a function of the *Fraction of Somatic Mutations *(X axis) in 27 cancer types for which there are a minimum of 4 points for fitting from at least 50 donors. The PanCancer plot is calculated merging all the cancer types together. The results of the fitting procedure are reported in Supplementary Table 2, Additional file 1.

We performed the recurrence analysis on each cancer type fitting the points calculated at different Mutation Recurrence threshold. The results in Figure 4 show different trend in 27 tumor types for which the regression curves can be calculated. Although in some cases the fitting is less accurate (THCA and PBCA), we can compare different tumor types estimating the fraction of somatic mutations covering 95% of the donors (Supplementary Table 2, Additional file 1). The smallest value corresponds to the Esophageal Adenocarcinoma (ESAD) for which we estimated somatic mutation rate of ~0.006%. The highest value is reported for the Pediatric Brain Cancer (PBCA) for which 75% the mutations are needed to cover 95% of the samples.

**Table 2:** Cancer genome databases and resources

Resource	URL	Ref
*Data repositories*		
CGHub	https://cghub.ucsc.edu	[34]
COSMIC	http://cancer.sanger.ac.uk	[132]
ICGC	https://dcc.icgc.org/	[133]
TCGA	https://tcga-data.nci.nih.gov/	[38]
		
*Cancer gene lists*		
Bushman Lab	http://www.bushmanlab.org/links/genelists	[45]
Cancer Gene Census	http://cancer.sanger.ac.uk/census	[134]
TumorPortal	http://cancergenome.broadinstitute.org/	[46]
Vogelstein List	http://goo.gl/4EmFG6 (Table S2A)	[9]
		
*Cancer genome resources*		
CaGe	http://mgrc.kribb.re.kr/cage/	[60]
Cancer3D	http://www.cancer3d.org/	[51]
Cancer Genomics Browser	https://genome-cancer.ucsc.edu/	[59]
cBioPortal	http://www.cbioportal.org/	[57]
DriverDB	http://driverdb.ym.edu.tw/DriverDB	[61]
IntOGen	http://www.intogen.org/	[62]
NCG	http://ncg.kcl.ac.uk/	[50]

In general, it is expected that heterogeneous cancer types show a large variety of recurrent somatic mutations, which is inversely proportional to the rate of decay of the complementary cumulative distribution (CCD). Thus, the analysis of recurrent mutations based on the calculation of the CCD can provide an estimation of the mutational heterogeneity of each cancer type (see Figure 4). Furthermore, the CCDs in Figures 3C and 4 provide an estimation of the maximum fraction of patients that can be recovered using a decreasing pool of recurrent variants.

**Figure 5:**
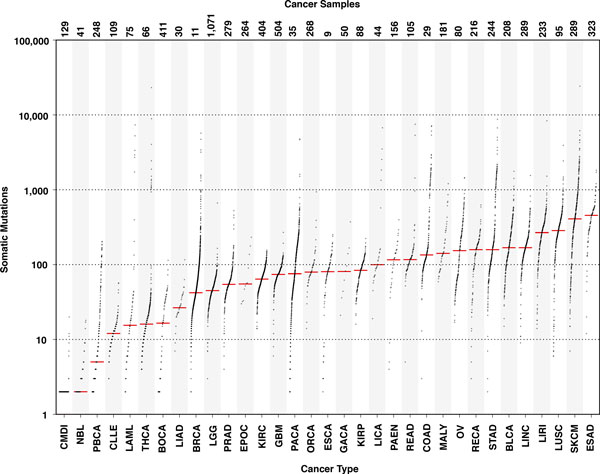
**Distribution of the number of somatic mutations in exonic regions for each donor in 33 cancer types**. The red line represents the median of the distribution for each cancer type. The plot is inspired from a recent paper [5]. The median values are reported in Supplementary Table 3, Additional file 1. Data from the same tumor type sequenced by different national consortiums are merged together.

Focusing on the exonic regions for each cancer type, we observed different number of somatic mutations per individual (Figure 5). The statistic shows a difference of ~2.5 orders in magnitude between the number of somatic mutations in Chronic Myeloid Disorders (CMDI) and Skin Cutaneous Melanoma (SKCM) for which their median values per individual in the exonic regions is 1 and ~400 respectively (Table 1). This result is consistent with the difference in the rate of decay of the CCDs observed for the two cancer types in Figure 4.

**Figure 6:**
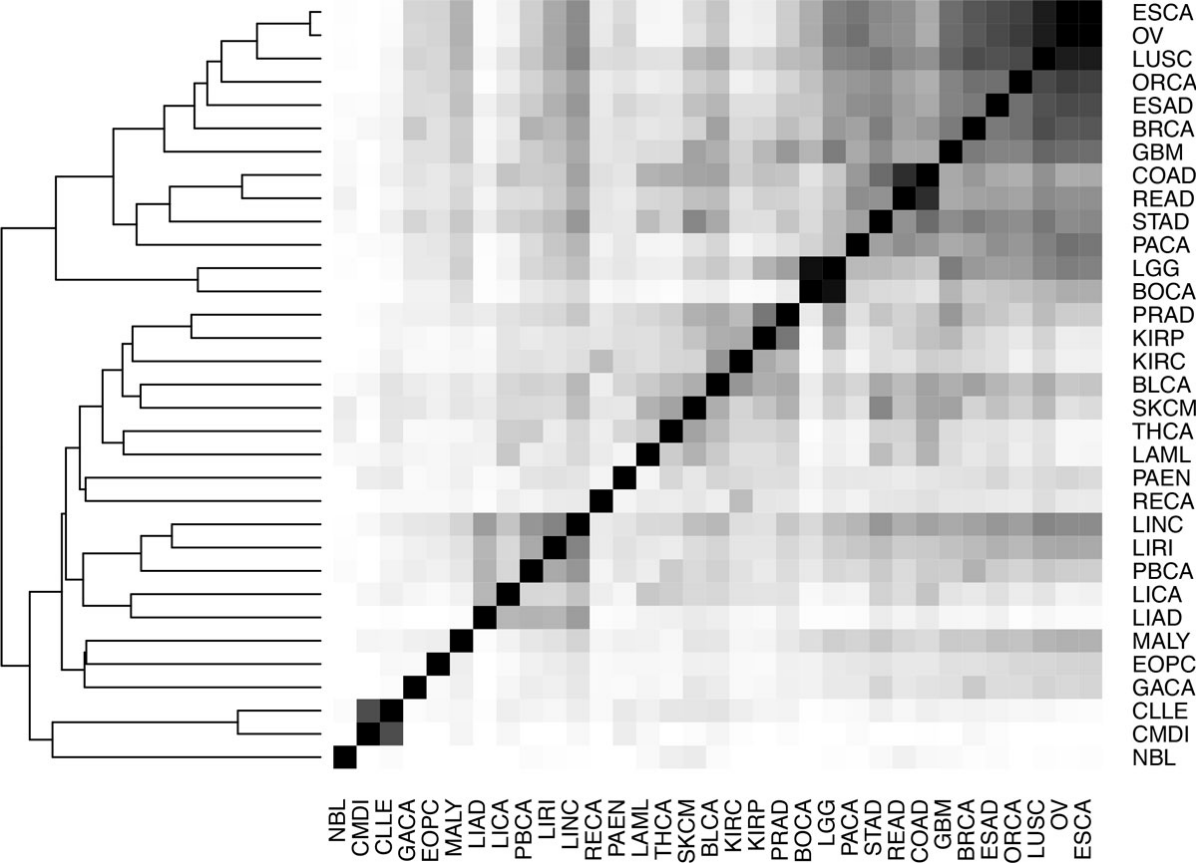
**Tumor cluster dendrogram derived from 33 cancer types using the hierarchical clustering approach**. The similarity between two tumors has been calculated using cosine similarity between gene-based vectors. Each element of the gene-based vector represents the number of donors in which a gene has at least one *Recurrent Somatic Mutation *(Section 3 in Additional file 1).

In addition, we can use the number recurrent mutations in the exonic regions to estimate the tumor type similarities. Using the subset of mutations repeated more than once in the whole dataset (PanCancer) we built a vector, in which each element represents the number of donors affected by a mutated gene. Only genes harboring the aforementioned recurrent mutations are considered. The gene-based vectors of each cancer type are then used to calculate the cosine similarity (Section 3 in Additional file 1). In Figure 6 we report the tumor dendrogram obtained from 33 cancer types. Broadly, based just on the similarity between the vectors of recurrent mutated genes we can cluster the tumors in two major subgroups of cancer. The figure shows on the first subgroup on the top-left side Colon Adenocarcinoma (COAD), and Rectum Adenocarcinoma (READ), in which high fractions of donors are mutated in APC, TP53 and KRAS, in the same cluster. In the second subgroup we observe 4 different liver cancers (LINC, LIRI, LICA and LIAD), with recurrent mutation in CTNBB1, in the same cluster.

These results confirm the hypothesis that recurrent somatic mutations, which represent only 1.7% of the whole PanCancer mutations, still retain meaningful information about the mutation profile of each cancer type that allows us to cluster them according to their similarity. Finally, it is noteworthy that filtering out non-recurrent somatic variants we significantly reduce the number putative driver mutations to a relatively small set of variants (~170,000), which are held by ~96% of the patients (see Table 1).

Although the analysis of somatic mutation data from multiple projects provides interesting insights about the genetic cause of tumor, we need to remember that data can have biases due to the use of different sequencing protocols and variant-calling procedures. These problems can be amplified with bias in sampling, significantly different size of the cohorts.

#### Curated databases and cancer gene lists

One problem that has been creating a constant roadblock in developing better tools and methods for cancer genome analysis is the lack of reference benchmark datasets of known cancer variants. The datasets discussed in this section are curated known collections of cancer variants that are used frequently as a substitute of a reference dataset.

The most popular collection of the somatic mutations in cancer is the Catalogue Of Somatic Mutation In Cancer (COSMIC). The last release of COSMIC (version 71, Sep 2014) contains ~2.1 million unique somatic variants detected in ~1 million tumor samples. An important manually curated resource made available through the COSMIC web page is the Cancer Gene Census list. Currently it contains 547 genes for which mutations have been causally implicated in cancer. Out of 547 cancer genes, 85% of them harbor only somatic mutations, 7% harbor only germline variants and the remaining 8% harbor both types.

Beside the Cancer Gene Census there are other lists of putative cancer genes identified using computational or expert-based approaches. One is a list of 125 cancer genes, reported in a publication by Vogelstein and collaborators [9]. The list comprises 54 oncogenes (43%) and 71 tumor suppressor genes (57%). A larger list of 2,125 genes collected by Bushman Lab [45] has been obtained as a union of 8 different datasets. More recently, an analysis of 21 cancer types was published. The authors prioritized the cancer causing genes according to their observed mutation frequency across multiple samples [46]. The final list thus obtained, combining all the cohorts, contains 146 genes, 64 of which are highly significantly mutated (44%), 49 significantly mutated (34%) and 32 near significant (22%). A better description of the available lists of putative cancer genes obtained from the analysis large cancer studies is provided in recent publications [47-49].

Other useful databases for the annotation of cancer genome are NCG [50] and Cancer3D [51]. The NCG 4.0 database contains 2,000 cancer genes, 537 of which have been experimentally validated and 1,463 statistically inferred from the analysis of candidate cancer genes in 3460 human exomes and genomes from 23 different cancer types. For each cancer gene, NCG provides information about duplicability, evolutionary origin, expression, functional annotation, interaction network with human proteins and microRNAs. Finally, protein structure is also a valuable piece of information for predicting the impact genetic variants [52] and effectiveness of drugs [53,54]. Cancer3D database collects ~275,000 somatic mutations mapped to ~15,000 proteins that correspond to more than 24,300 structure from the Protein Data Bank [55]. The idea is to study the impact of missense somatic mutations on protein three-dimensional structure. The Cancer3D database includes predictions from e-Drug and e-Driver, two recently developed tools for predicting sensitivity to 24 anticancer compounds and cancer driver proteins [56].

#### Resources for the visualization and analysis of cancer genome data

In the last few years, several web portals have been developed for the visualization and analysis of all kinds of data in cancer genome studies. Among the most used is the cBioPortal maintained by the Memorial Sloan Kettering Cancer Center [57,58]. The cBioPortal can be queried either using the web interface or through programmable application programming interface (API). The cancer data displayed by the cBioPortal are extracted from 18,469 samples from 80 cancer genome studies. To facilitate the submission of queries, cBioPortal implements the Onco Query Language (OQL) for selecting and defining genetic alterations for any subset of data. The user interface allows searching for somatic mutations or copy number alteration across different samples and cancer studies selecting variations occurring in user-defined gene list or in 22 predefined groups cancer-specific genes. When available, expression level from RNA-Seq experiments, methylation and protein phosphorylation data can also be retrieved.

A possible alternative to cBioPortal is the Cancer Genome Browser [59]. The Cancer Genome Browser allows extraction and visualization of information from 574 datasets. Among these two resources, cBioPortal provides better integration with pathways data and the Cancer Genome Browser allows better visualization of clinical data.

Other three interesting resources for the analysis of cancer genome data are the Cancer Gene annotation system for Cancer Genomics (CaGe) [60], the DriverDB [61] and the Integrative Oncogenomics Cancer Browser (IntOGen) [62]. CaGe is a cancer genome annotation system for the classification of candidate genes from cancer genome studies, using either previously reported or novel categories of cancer genes, providing insights about the underlying carcinogenic mechanisms through pathway analysis. DriverDB incorporates data from 6,079 exome-sequencing experiments from 33 cancer studies. It integrates annotation databases and eight bioinformatics algorithms for detecting driver genes and mutations. Finally, Integrative OncoGenomics (IntOGen) is a web available resource integrating data from 6,792 genomes/exome sequencing experiments from 28 cancer types (release 2014.12) [63]. It collects and analyses somatic mutations in a large set of tumor samples to identify putative cancer driver genes. The prediction of putative cancer driver genes is performed by OncodriveFM [64], which has been developed by member of the same group (see next section). The web interface allows browsing data by gene name, cancer type, sites and projects, reporting the frequencies of mutation at gene and genomic location InOGen also allows the study of genomic alterations in cancer in the contest of pathways [65].

The URLs of the cancer genome data repositories, the cancer gene lists and resources for cancer genome analysis are summarized in Table 2.

### Computational methods for cancer genome interpretation

The analysis of the cancer genome is a challenging task from both the experimental and computational point of views. A recent review, in addition to providing an exhaustive overview of the available tools for the detection of somatic mutation from sequencing data, also highlighted these challenges [11]. The lack of a perfect pipeline for the detection of genetic variants and the computational analysis of the data remains one of the major bottlenecks in the field. The current computational methods for the interpretation of variants in cancer genome have been developed to address the following issues: i) Detection of recurrent somatic mutations and cancer driver genes; ii) Prediction of driver variants and their functional impact; iii) Estimate the impact of multiple variants at network and pathway level; iv) Differentiate subclonal populations and their variation patterns. In this section we describe a selection of available computational tools for addressing the four issues listed above.

#### Detection of cancer driver genes

In general, the insurgence of cancer is accompanied with an accumulation of somatic mutations. However, this does not imply that all mutations are of equal importance in the oncogenesis and cancer progression. Rather, driver somatic mutations exert a selective advantage to cancer cells. In contrast, passenger mutations are abundant but do not confer any selective advantage. Driver variants are present in a small fraction that may vary in different cancer types [66,67]. Thus, an obvious approach for the selection of cancer driver genes involves the analysis of recurrent somatic mutations. Following this idea, several methods - such as MuSiC [68], MutSigCV [69] DrGaP [70] and Simon's tool [71] - have been developed to prioritize cancer driver genes using different statistical models for the estimation of a background mutation rate.

The Mutational Significance in Cancer (MuSiC) uses sequence-based input to establish correlations among mutation sites, affected genes and pathways, and to discriminate abundant passenger mutations from significant mutation events. This method aims to identify significantly mutated genes with respect to a background mutation rate. MuSiC has been tested on a set of 316 ovarian cancer samples and it was able to detect 12 significantly mutated genes [68]. MutSigCV is the newest version of the "Mutation Significance" algorithm that uses gene specific background mutation rates including mutation events in gene covariates. The information from known co-varying genes is important for estimating the background mutation rates in the genomic regions where few mutation events are observed. MutSigCV takes an input list of mutation from different samples and builds a model gene-dependent background mutation rate estimated using clusters of genes. MutSigCV has been used to analyze 3,083 tumor-normal pairs, and it was able to discover strong differences in mutation frequency and spectrum across 27 cancer types, providing interesting insights about the etiology of the disease [69]. DrGap [70] is a statistical framework for identifying driver genes and signaling pathways in cancer genome-sequencing studies. This approach integrates biological knowledge of the mutational process in tumors and uses a heuristic strategy to optimize the mixture proportion of chi-square distribution of likelihood ratio test (LRT) statistics. This approach improves the accuracy and sensitivity of the prediction of driver genes avoiding zero estimation of the driver mutation rates due to the small probability of observing any mutation in the available samples. Simon's tool [71] calculates the background mutation rate by accounting for the functional impact of mutations on proteins, variation in background mutation rate among tumors, and the redundancy of the genetic code. Using this algorithm, the authors reanalyzed 623 candidate genes in 188 non-small cell lung tumors and identified 28 driver genes, 6 of which were novel [72].

Alternative approaches have been implemented in ActiveDriver [73], OncodriveFM [64], OncodriveCLUST [74] and ContrastRank [75]. ActiveDriver is able to detect significantly mutated functional sites in cancer genome providing an analysis of the somatic mutations associate to phosphorylation signaling. Indeed, the predictive model has been calculated considering the frequency of somatic variants from ~800 cancer genomes in proximity of ~74,000 phosphorylation sites and 469 kinase domains [73]. The method was able to identify candidate genes, protein complexes and kinases involved in cancer. OncodriveFM [64] is a method for the detection of putative cancer driver genes or gene modules. It computes the functional impact of variants using three established computational approaches (SIFT [76], PolyPhen2 [77] and MutationAssessor [78]). OncodriveFM prioritizes putative cancer driver genes calculating the distribution of the predicted functional impact scores for several variants across tumor samples and its deviation from the null model. The bias towards the accumulation of variants with high functional impact is used detect candidate driver genes. Similar approach has been used to prioritize cancer-associated pathways. OncodriveCLUST has been developed to identify significant bias towards somatic mutations clustered within the same protein. The background model for prioritizing genes is calculated using the rate on silent mutations, which are not assumed to be under positive selection. OncodriveCLUST cluster scores have been calculated using ~240,000 mutations in ~9,500 genes from COSMIC database and has been tested of on four TCGA datasets. This approach provides variable background mutation rate for each gene and it detect recessive cancer genes not identified using OncodriveFM [74].

Finally, we also highlight ContrastRank [75], which is a new method for the prioritization of putative impaired genes in cancer. With respect to previously developed methods, ContrastRank evaluates the background mutation rate using the maximum value between the mutation rates of each gene in 1000 Genomes [79] and normal TCGA samples. For the calculation of the score the methods only considers mutation with allele frequency in 1000 Genomes lower than 0.5% and uses the complement of the cumulative binomial distribution to rank cancer associated-genes. The method has been tested on TCGA from three types of adenocarcinomas. In addition, the Cancer Census, Vogelstein and Bushman cancer gene lists (see Table 2) have been used to assess the quality of the prioritization method. ContrastRank was also used for calculating an exome-based score to discriminate between TCGA normal and tumor TCGA samples resulting in high level of accuracy.

#### Predicting the impact of non-synonymous variants in cancer

During the last decades several methods have been developed to predict the impact of non-synonymous single nucleotide variants (nsSNVs) at structural [80] and functional levels [2]. In particular, the algorithms for predicting the functional effect of missense variants estimate the probability that a mutation is disease-associated or functionally deleterious. Although the relationships between molecular state and disease are complex and are far from being completely understood, the pathologic effect resulting from amino acid substitution is commonly estimated by predicting its functional impact. Most of the available algorithms are based on the evidence that functionally important protein sites are under purifying selection [81]. Therefore, the majority of disease-causing variants should occur in conserved regions that can be detected by evolutionary analysis [82]. Using this approach many classifiers have been implemented to predict whether a nsSNV has any functional impact. The most famous methods for estimating the impact of genetic variants are SIFT [76] and PolyPhen [77] whose predictions are already embedded in many variant annotation pipelines. Recently, more sophisticated methods, which exploit additional structural and functional information, have been developed [19,83-85]. Recent advances in the field are represented by the implementation of consensus-based algorithms [86,87] and a general approach that is able to predict the impact of variants in non-coding regions [81,88].

Although most variant effect predictor reached an adequate level of accuracy, their predictions do not provide information about the possible phenotypic effect. This problem has been partially addressed with the development of a new class of disease-specific predictors trained on a subset of mutations with defined phenotypic effect. In particular, several methods have been developed for discriminating between passenger and cancer driver mutations [78,89-93]. Among them, CanPredict [91] has been the first algorithm for predicting cancer-causing mutations. The method uses a conservation measures from PFAM [94] Hidden Markov Models and functional information encoded through Gene Ontology terms. Similar strategy has been implemented in support vector-based approach (Dr. Cancer) that uses sequence profile and cancer-specific functional terms [89]. Dr. Cancer has been tested on 3,163 cancer-drive mutations from 74 proteins. The results show that cancer-related proteins are enriched for particular Gene Ontology terms that can be used to discriminate between cancer and other phenotypes. The same dataset was previously used to train CHASM a random forest-based algorithm [90]. CHASM was tested on ~600 missense mutations in glioblastoma multiforme achieving better performance than PolyPhen and previously developed cancer specific approaches. More recently, new methods such as MutationAssessor [78], TransFIC [93] and FATHMM [92] have been optimized to detect cancer driver variants. MutatationAssessor uses evolutionary information patterns calculating an entropy-based functional impact score from homolog and paralog proteins. The method trained on ~19,000 disease associated variants has been tested on 10,000 mutations in COSMIC database prioritized according to their recurrence and multiplicity. The authors estimated that ~5% of the cancer-relevant mutation involves a change in the protein function rather than standard loss and gain of function mechanism events. In TransFIC approach the prediction of cancer drivers mutation has been tuned considering subset of variants associated to the same Gene Ontology term and selecting a variable threshold for discriminating driver from passenger mutations. The performance of the optimized functional impact score has been tested on subset of the COSMIC mutations classified according to their recurrence. The results show that groups of protein with different function, posses distinct baseline tolerance to deleterious mutations. Finally, FATHMM is a Hidden Markov Model based algorithm using protein domain information that has been optimized for predicting cancer causing mutations. The method, tested on previously collected datasets result in improved performances with respect to previously developed methods. Although the algorithms based on functional information (Gene Ontology terms/ protein domains) achieve better results than standard conservation-based approaches, a fair testing procedure is more difficult because the predictions can be biased toward more abundant functional classes.

#### Analysis of cancer gene pathways and networks

The accumulation of somatic mutations during the lifespan is the main cause of cancer. Several identified somatic mutations occur in genes involved in many signaling, regulatory and metabolic pathways. Indeed mutated genes such as TP53 and PI3KCA are hubs in pathways and interaction networks which control cell proliferation, growth and apoptosis [95,96]. In addition, recent sequencing studies [4,38] revealed that cancer driver genes tend to cluster within a limited number of essential pathways, and rarely mutations on genes in the same pathway co-occur in the same patient. This mutually exclusive genomic events have been observed, for example, in the case of *BRAF *and *KRAS *(involved in RAS/RAF pathway) in colorectal cancer [97], *APC *and *CTNNB1 *(involved in beta catenin pathway) [98], *EGFR *and *KRAS *in lung adenocarcinomas [99], *TP53 *and *MDM2 *in many different cancer types [100].

From these observations, it is evident that the analysis of genomic variations across gene pathways and networks is important to understand the combinatorial effect of the mutations and explain the disease mechanism. Pathway and network analysis can be performed using previously annotated gene pathways or testing alternative routes from gene interaction networks. Reference databases collecting information about gene pathways are the Kyoto Encyclopedia of Genes and Genomes Kyoto Encyclopedia of Genes and Genomes (KEGG) [65] and the Molecular Signatures Database (MSigDB) [101]. Biological interaction networks mainly consist of protein-protein interaction data, which are collected in databases such as IntAct [102] and iRefIndex [103]. Another important database is the Reactome, which aggregates data on protein-protein interaction networks and gene pathways [104].

Recently, several methods for the analysis of cancer gene pathway have been developed [105-115]. Few examples are PathScan [114], which is an annotation-based approach, and HotNet2 [115], MDPFinder [107], MEMo [105], and Dendrix [113] which are able to identify driver pathways. PathScan [114] is a probabilistic model that takes into account the length of the genes and differences in their mutation rates under the null hypothesis. The method combines single-sample p-values using an integral approximation that estimates a pathway-specific overall p-value. PathScan represents an alternative to previous approaches, which reduce a gene set into a unique gene simply combining the total mutations and mutable positions of each gene. PathScan was applied to the analysis of data from lung adenocarcinoma sequencing project to find significantly mutated 129 KEGG pathways. The results show that PathScan was able to identify significant pathways detected in previous studies. In addition PathScan found the focal adhesion pathway to be significant in agreement with previous expression studies on prostate and ovarian cancers [114]. HotNet2 [115], has been recently developed for identifying significantly mutated groups of interacting genes from large cancer sequencing studies. It uses a heat diffusion model that encodes both the topology of the network and the significance of the observed frequencies of each mutated gene. HotNet2 has been tested on 3,281 samples from 12 cancer types in TCGA studies. In this analysis it was able to identify 16 significantly mutated subnetworks that comprise well-known cancer signaling pathways. Among them, the well-known TP53, phosphoinositide 3-kinase (PI3K), NOTCH and receptor tyrosine kinases (RTK) signaling pathways.

Although annotation-based methods are able to prioritize important cancer pathways, they present some limitations. The main limitations are the incompleteness of pathway databases and the presence of multiple overlapping gene sets across pathways. Thus, more general methods for detecting significantly mutated gene sets use information from biological interaction networks. The Mutual Exclusivity Modules in Cancer (MEMo) algorithm [105] integrates copy number alteration and mutation data and maps them into biological networks. The method uses correlation analysis and statistical tests to identify network modules of genes recurrently altered across a set of tumor samples, participate in the same biological process, and alteration events are mutually exclusive. MEMo has been tested on a set of ~400 samples from glioblastoma and ovarian cancer. In the first cancer study the method was able to identify important signaling modules such as p53 and PI(3)K. In ovarian cancer, it detected mutually exclusive variation events between BRCA and genes in the Rb module.

The De novo Driver Exclusivity (Dendrix) algorithm [113] is a tool for discovery of mutated driver pathways in cancer using only mutation information from the cancer samples. The method introduces the concepts of coverage and exclusivity to distinguish group of genes with driver mutations from set of genes with passenger mutations. The Dendrix algorithm, which has been applied to the analysis of different cancer types in TCGA study, was able to identify 8 mutation groups mutated in 94% of patients from 17 cancer types and groups of mutually exclusive genes in lung adenocarcinoma and glioblastoma multiforme. Similarly, MDPFinder [107] identifies de novo mutated driver pathways from mutation data solving the maximum weight submatrix problem. The authors implemented an exact method based on binary linear programming and genetic algorithm to combine mutation and expression data. MDPFinder has been tested on a set of ~500 samples from head and neck squamous cell carcinomas glioblastoma and ovarian carcinoma. The results show that the integrative model based on mutation and expression data was able to identify biologically relevant gene sets detected in previous studies [107].

Finally, PARADIGM[116] estimates patient-specific genetic activities incorporating curated gene interactions from the NCI pathway interaction database (PID) [117]. The method, that can use many types of omics data, represents a gene by a factor graph with associated known activity and expression. PARADIGM, tested on breast cancer and glioblastoma samples, was able to identify altered activities in cancer-related pathways with less false-positives than a previous developed method.

An interesting approach, which applies network analysis to the study of the cancer genome, is the Network-Based Stratification (NBS) method [118]. NBS is an algorithm for the classification of cancer subtypes that clusters patients with somatic mutations in similar gene network regions. More in details, the NBS represents each patient with a profile of somatic mutated genes mapped on the human gene interaction network. After a smoothing procedure, the patients are clustered using a non-negative matrix factorization-based approach. The method performs a second clustering procedure to find subgroups of patients frequently co-stratified after random sampling. NBS has been tested on a set of ~1,000 samples from ovarian, uterine endometrial and lung tumors and used to determine relationship between cancer subtypes and patient survival.

#### Classification of cancer subclonal variants

Tumor progression is an evolutionary process which starts from a single cell and results in the selection of more aggressive subclones [119]. The presence of different cell populations affects the accurate detection of somatic mutations in cancer sample. Although all subclones in a sample have the same origin, they are present in different proportions and have heterogeneous patterns of somatic mutations. Thus, somatic mutations in cancer cells present in small proportion are more difficult to detect because correspond to variations supported by a low number of reads, further reduced by impurity in the tumor sample. In a recent work [13], Ding and colleagues estimated that approximately 340X coverage is needed for detecting (with 99% of chance) at least 3 reads when a heterozygous variant is present in a 5% subclonal population.

**Figure 7:**
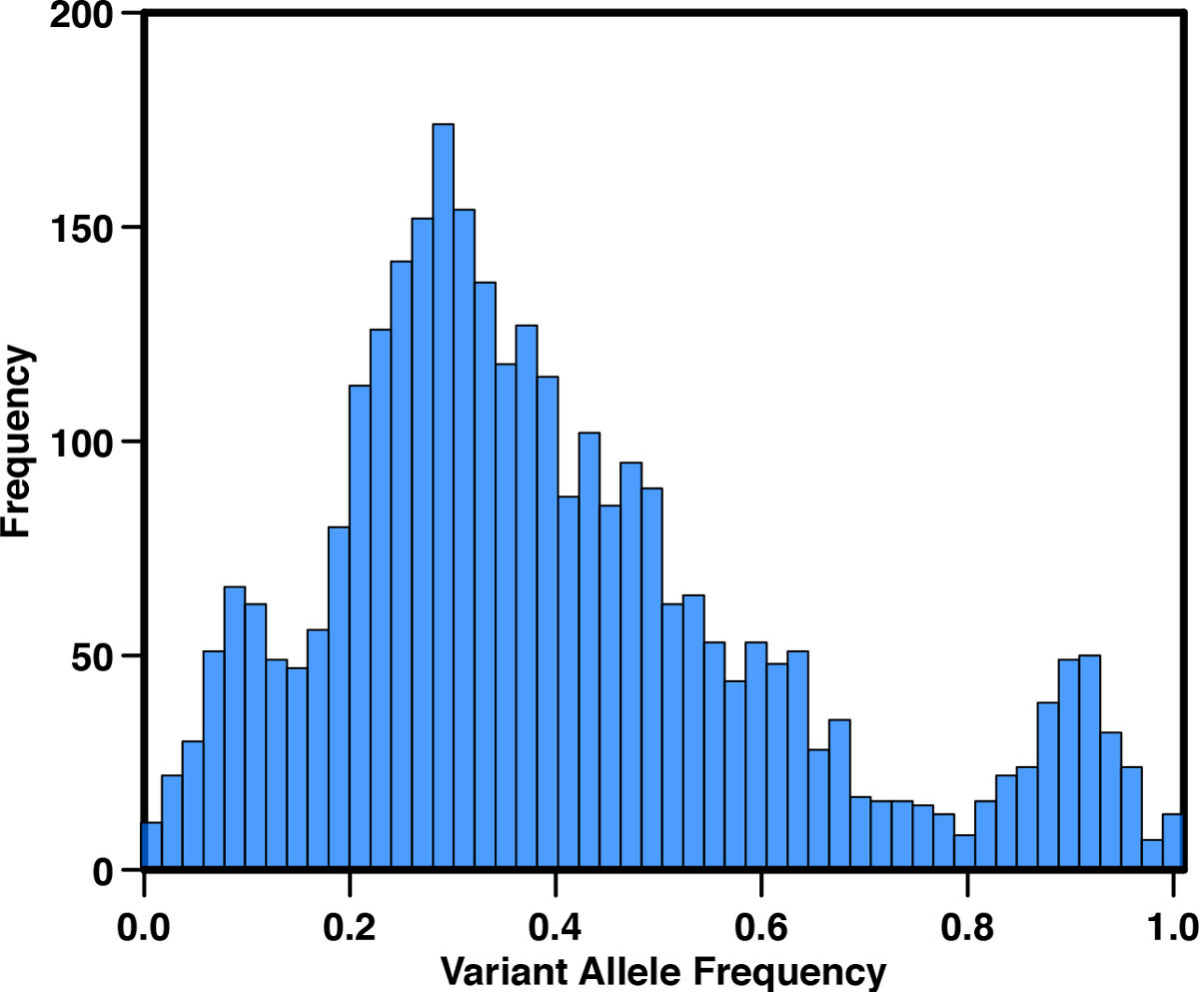
**Distribution of the Variant Allele Fraction (VAF) of somatic mutations in one sample of lung adenocarcinoma from the TCGA study**.

A possible solution to this issue lies in single-cell sequencing for revealing diversities in the pattern of somatic mutations within a tumor [120,121]. Although this technique provides a better characterization of the mutations occurring in each subclonal population, the approach is affected by errors introduced during the amplification process. Furthermore, single-cell experiments need to be performed on multiple tumor cells to have a general overview of the mutation pattern. The presence of subclones in the cell population can be detected analyzing the distribution of reads or variant allele fraction for the somatic mutations. If the distribution is multimodal, the presence of subclonal populations is expected. Figure 7 shows the distribution of variant allele fraction (VAF) in a lung adenocarcinoma cancer sample from TCGA. Although the distribution of variant allele fraction provides an indication about the clonal architecture, the analysis of this data is affected by sample impurity and copy number alterations. In the last few years many computational tools have been developed to estimate the presence of subclonal populations [122-127], some of them are discussed below.

ABSOLUTE [122] uses data from copy number variations for optimizing models of recurrent cancer karyotypes and expected allelic fraction values for somatic SNVs. These models are then used to re-extract information about the absolute cellular copy number of local DNA segments and the number of mutated alleles for somatic SNVs. The ABSOLUTE algorithm has been applied to the analysis of copy-number profiles from 3,155 cancer samples, identifying recurrent genome doubling events that influence tumor progression. ABSOLUTE was also used for the analysis of exome sequencing data from 214 ovarian carcinoma tumor-normal pairs. The method was able to identify large subclone populations with predominant somatic mutations and small subset of subclones with heterozygotes mutations in tumor suppressor genes TP53 and NF1 and in a candidate tumor suppressor gene CDK12.

Alternative methods [124,125] use phylogenetic approaches to study the evolution of tumor cells. For example, PyClone [125] assumes that clonal population follows a perfect and persistent phylogeny. Under this assumption, for which each somatic mutation only happen once in the evolutionary history and not reverse mutation are allowed, subclonal cells can be identified and their prevalence estimated. PyClone uses a Bayesian clustering algorithm to group sets of somatic mutations belonging to the same cluster of subclonal cell accounting for allelic imbalances introduced by copy-number alterations and normal-cell impurity. The method has been tested on a simulated dataset produced from mixtures of DNA extracted from four 1000 Genomes Project sample and four spatially separated samples from a primary, untreated high-grade ovarian tumor. The results show that PyClone outperforms two genotype-naïve methods using binomial and infinite beta-binomial mixture models. Similarly, PhyloSub [124] infers the phylogeny and genotype of the major lineages in the clonal population calculating the Bayesian prior over the trees clustering the SNVs. A sampling procedure is use to find the optimized the join posterior distribution with higher probability to generate the observed frequencies of somatic mutations. PhyloSub has been tested using deep exome sequencing data from acute myeloid leukemia and chronic lymphocytic leukemia. The results show that PhyloSub is able to identify both linear and branching subclonal lineages.

A recent publication [123] presents a novel combinatorial approach based on binary partition tree (BPT) to model the mechanism of clonal expansion in tumor and estimate the subpopulations of tumor using the variant allele frequencies of somatic mutations. The authors demonstrated that finding a BPT is a NP-complete problem, and derived an approximation algorithm for an optimized version of the problem. Finally they implemented a recursive approach that finds the solution of the optimized BPT problem in a polynomial time. The developed algorithm can detect errors in the estimation of the variant allele frequencies of somatic mutations, which cannot be correctly estimated because of the admixture of normal cells in the tumor sample. The performance of BPT algorithm has been tested on simulated and real cancer data showing it generates more consistent results, and it is faster than previously developed methods.

The methods for the detection of genetic heterogeneity in cancer can be also used to detect subclonal mutation conferring drug resistance. This idea has been investigated using cloneHD [126], a new subclonal reconstruction algorithm optimized using both information about somatic mutations and correlated changes generated by copy-number changes. The method has been applied to the analysis of sequencing data from time-resolved samples from breast cancer and of chronic lymphocytic leukemia. The results demonstrate that cloneHD can be a valuable tool for tracking cancer development and monitoring the response of a patient to therapy regimens.

The URLs of the tools describe above for the analysis of the cancer genome are summarized in Table 3.

**Table 3:** Computational methods for cancer genome interpretation

Method	URL	Ref
*Cancer gene prioritization*		
ActiveDriver	http://individual.utoronto.ca/reimand/ActiveDriver/	[73]
ContrastRank	http://snps.biofold.org/contrastrank/	[75]
DrGaP	https://code.google.com/p/drgap/	[70]
MuSiC	http://gmt.genome.wustl.edu/packages/genome-music/	[68]
MuSigCV	http://www.broadinstitute.org/cancer/cga/mutsig	[69]
OncodriveCLUST	http://bg.upf.edu/oncodriveclust	[74]
OncodriveFM	http://bg.upf.edu/oncodrivefm	[64]
Simon's tool	http://linus.nci.nih.gov/Data/YounA/software.zip	[71]

*Cancer variant annotation tools*		
CanPredict	http://goo.gl/UK9lbv	[91]
CHASM	http://wiki.chasmsoftware.org/	[90]
DrCancer	http://snps.biofold.org/drcancer/	[89]
FATHMM	http://fathmm.biocompute.org.uk/cancer.html	[92]
MutationAssessor	http://mutationassessor.org	[78]
TransFIC	http://bg.upf.edu/transfic/	[93]
		
*Pathway and network analysis*		
Dendrix	http://compbio.cs.brown.edu/projects/dendrix/	[113]
HotNet2	http://compbio.cs.brown.edu/projects/hotnet2/	[115]
MDPFinder	http://zhangroup.aporc.org/ShiHuaZhang	[107]
MEMo	http://cbio.mskcc.org/memo	[105]
PathScan	http://genome.wustl.edu/software/pathscan	[114]
PARADIGM	http://sbenz.github.com/Paradigm	[116]
*Classification of tumor subclonal variants*		
ABSOLUTE	http://www.broadinstitute.org/cancer/cga/absolute	[122]
BTP	http://compbio.cs.brown.edu/projects/btp/	[123]
CloneHD	https://github.com/andrej-fischer/cloneHD/	[126]
PhyloSub	https://github.com/morrislab/phylosub/	[124]
PyClones	http://compbio.bccrc.ca/software/pyclone/	[125]

### Concluding remarks and future perspectives

In this review we provide an overview of the challenging topics in the analysis of cancer genome. We mainly focused on the characterization of single nucleotide variant, which is by far the most common type of genetic variation. In particular, we provided a summary of the most important cancer genome data available online and described a selection of the available computational tools for cancer genome interpretation.

Although several algorithms have been developed, the problem of cancer genome interpretation is far from being solved. The progress in the field is limited by many factors mainly associated with (a) the intrinsic complexity of the problem (b) technical limitations, and (c) ethical issues.

The complexity of the problem mainly comes from the huge number of somatic variations present in the in each tumor sample and our inability to select driver and clinically actionable variants. The technical limitations are also affecting the detection of genetic variations present in a smaller fraction of subclones. The third important issue is the restricted access to the data for protecting the privacy of the individual.

It is expected that in the near future most of the limitations will be overcome by the development of more accurate computational tools and experimental approaches which will play an important role in the understanding the relationship between genotype and phenotype in cancer. In particular, brute force sequencing initiatives will result in a better mapping of the functionally important regions in the genome and experimental approaches, such as CRISPR/Cas [128] will provide the opportunity to extensively test the functional impact of genetic variants. In addition, an improvement of the single cell sequencing technology will allow better characterization of the progression of tumor and definition of the pattern of mutations in more aggressive subclonal cells.

The integration of more accurate data will have an impact on the development of more accurate computational tools. Indeed, the limited ability to score the performances of currently available algorithms can be addressed by collecting standardized benchmark sets from high-quality experiments. An important component for speeding up this learning process involves the implementation of more effective policies for data sharing. Although major efforts have been made by the TCGA, ICGC and other cancer consortium, the procedures for releasing sequencing data need to be optimized. Standard protocols for extracting and reporting the data are also required for efficient data analysis. Information about the germline variants present in sequenced patients should be made available for validating hypothesis about the tumor predisposition. Furthermore, it will be extremely important to develop appropriate de-identification procedures [129] and the promotion of informed consent policies for improving the effective usage of genotype/phenotype databases [130,131].

## Competing interests

The authors declare that they have no competing interests.

## Authors' contributions

RT and EC carried out the analysis of ICGC data. RT, MKB and EC wrote the manuscript and approved its final version.

## Supplementary Material

Additional file 1 **Supplementary materials including supplementary tables**. Click here for file

## Declarations

Publication costs for this article were funded by EC's start-up funds from the Department of Pathology at the University of Alabama, Birmingham.

This article has been published as part of *BMC Genomics *Volume 16 Supplement 8, 2015: VarI-SIG 2014: Identification and annotation of genetic variants in the context of structure, function and disease. The full contents of the supplement are available online at http://www.biomedcentral.com/bmcgenomics/supplements/16/S8.
