# Supplementary material for: Computational methods and resources for the interpretation of genomic variants in cancer
Source: BMC Genomics. 2015 Jun 18;16(Suppl 8):S7. doi: 10.1186/1471-2164-16-S8-S7 (PMC4480958; doi:10.1186/1471-2164-16-S8-S7)
Supplement: Additional file 1 — Supplementary materials including supplementary tables. [file 1471-2164-16-S8-S7-S1.pdf]

Supplementary Material

Computational methods and resources for the interpretation of genomic variants in cancer

Rui Tian[1], Malay K Basu[1,2] and Emidio Capriotti[1,2,3].*

[1] Division of Informatics, Department of Pathology, [2] Department of Clinical and Diagnostic Sciences and [3] Department of Biomedical Engineering,
University of Alabama at Birmingham, 619 19th St. South, 35249 Birmingham, AL (USA)

*Corresponding author: Emidio Capriotti, emidio@uab.edu

1. Dataset

To provide an overview of the somatic mutation data available, we analyzed the variants from the International Cancer Genome Consortium (ICGC) data portal (https://dcc.icgc.org/) release 17 (September 2014). We collected the simple somatic variants corresponding to 43 different cancer projects and after manual inspection we removed the data from Acute Lymphoblast Leukemia (ALL-US), which consist of only 2 samples. The final list of 42 cancer projects is reported in Supplementary Table 1. In our analysis, we merged data from the same cancer types coming from different projects. Thus, our dataset consists of 33 unique cancer types. Finally, we build a dataset, referred to as PanCancer, by pooling all the data from the previous 42 cancer projects.

2. Somatic mutation recurrence analysis

A large fraction of somatic mutations observed in cancer is passenger and does not have significant impact on the progression of the disease. In contrast, a small percentage of mutations, defined as drivers, increase the fitness of tumor cells. On average, it is expected that driver mutations are more recurrent than passengers across different cancer samples. For this reason, we analyzed all the 33 cancer types to study the recurrence of mutation events.
In this paper, we use the following definitions:

i. *Recurrent Somatic Mutation*: a variation that is observed at least in two donors of our dataset.
ii. *Mutation Recurrence*: the number of samples in the dataset in which a specific somatic mutation is observed. A *Recurrent Somatic Mutation* has *Mutation Recurrence* equal or bigger than to 2.
iii. *Fraction of Somatic Mutations*: it represents the portion of somatic mutations with *Somatic Mutation Recurrence* equal or higher than a given threshold. A particular case defined, as *Fraction of Recurrent Mutations,* is the number of somatic mutations observed at least in two donors divided by the total number of unique mutations.
iv. *Fraction of Donors*: it is calculated as the fraction of donors in which is observed at least for one somatic mutation with *Mutation Recurrence* equal or higher than a given threshold.

These values can be calculated for each cancer type separately or for the PanCancer dataset. In the latter case, the number of *Recurrent Somatic Mutations* increases because the mutations can also occur in two donors affected by different cancer types. To show this

difference, in Fig. 3 of the manuscript, we present the complementary cumulative distributions (CCDs) observed when cancer types are considered separately or together.
Although the values defined above are affected by the consistency (i.e., biases due to batch effects) of the dataset, it is still useful to estimate the expected *Fraction of Donors* that can be recovered using a subset of currently identified recurring variants. Thus, the recurrence analysis presented in this paper consists of plotting the *Fraction of Somatic Mutations* and *Fraction of Donors* at different *Mutation Recurrence* thresholds. In Fig. 4 we report the CCDs obtained for 27 cancer types with at least 50 donors and for which at least 4 points are available (therefore, NBL, CLLE, LICA, EOPC, LIAD and GACA are excluded). To estimate the trend of the curves, the points have been fitted using the following equation

$$Y = \frac{1}{A - BX^{C}} \quad [1]$$

Where *B=1-A*. This equation has been used to estimate the fraction of somatic variants that recurs in 95% of the donors (see Supplementary Table 2).

3. Exonic mutations and gene-based analysis

For the analysis of the ICGC data, we also focused on subset of variants in exonic region. To select this subset of variants we consider only the somatic mutations with assigned Ensembl gene code and discarded all the upstream, downstream and intronic mutations. Using this subset of somatic mutations in exonic regions we calculated the number variants corresponding to each donor and the relative distribution for 33 cancer types reported in Fig. 5 of the manuscript.
In addition we used a subset of 62,206 exonic *Recurrent Somatic Mutations* from the PanCancer to calculate a feature vector for tumor type similarity comparison. Thus, each cancer type can be described with a vector of 17,381 elements that correspond to the total number of genes with at least one exonic *Recurrent Somatic Mutation*. Each element represents the number of donors with the corresponding gene affected by a *Recurrent Somatic Mutation*.
Two cancer *A* and *B,* described by the vectors V_A and V_B, are compared using the cosine similarity that is defined as follows:

$$\cos(\theta) = \frac{V_A \cdot V_B}{\|V_A\| \, \|V_B\|} \quad [2]$$

The values of exonic *Recurrent Somatic Mutation*, the affected genes and donors for each cancer type are reported in Supplementary Table 3.
The cosine similarity measure is used to build the dendrogram of tumors reported in Fig. 6. The dendrogram is obtained using a hierarchical clustering algorithm implemented in the heatmap.2 function in R.

Supplementary Table 1

Project Code	Cancer Type	Origin	Donors
BLCA-CN	Bladder Cancer	CN	103
BLCA-US	Bladder Urothelial Cancer	US	130
BOCA-UK	Bone Cancer	UK	66
BRCA-UK	Breast Triple Negative/Lobular Cancer	UK	117
BRCA-US	Breast Cancer	US	954
CLLE-ES	Chronic Lymphocyclic Leukemia	ES	109
CMDI-UK	Chronic Myeloid Disorders	UB	129
COAD-US	Colon Adenocarcinoma	US	216
EOPC-DE	Early Onset Prostate Cancer	DE	11
ESAD-UK	Esophageal Adenocarcinoma	UK	95
ESCA-CN	Esophageal Cancer	CN	88
GACA-CN	Gastric Cancer	CN	9
GBM-US	Brain Glioblastoma Multiforme	US	268
KIRC-US	Kidney Renal Clear Cell Carcinoma	US	404
KIRP-US	Kidney Renal Papillary Cell Carcinoma	US	156
LAML-KR	Acute Myeloid Leukemia	KR	75
LGG-US	Brain Lower Grade Glioma	US	279
LIAD-FR	Benign Liver Tumour	FR	30
LICA-FR	Liver Cancer	FR	29
LINC-JP	Liver Cancer	JP	244
LIRI-JP	Liver Cancer	JP	208
LUSC-KR	Lung Cancer	KR	111
LUSC-US	Lung Squamous Cell Carcinoma	US	178
MALY-DE	Malignant Lymphoma	DE	44
NBL-US	Neuroblastoma	US	41
ORCA-IN	Oral Cancer	IN	50
OV-AU	Ovarian Cancer	AU	93
OV-US	Ovarian Serous Cystadenocarcinoma	US	88
PACA-AU	Pancreatic Cancer	AU	392
PACA-CA	Pancreatic Cancer	CA	112
PAEN-AU	Pancreatic Cancer Endocrine neoplasms	AU	35
PBCA-DE	Pediatric Brain Cancer	DE	248
PRAD-CA	Prostate Adenocarcinoma	CA	9
PRAD-UK	Prostate Adenocarcinoma	UK	21
PRAD-US	Prostate Adenocarcinoma	US	234
READ-US	Rectum Adenocarcinoma	US	80
RECA-CN	Renal Cancer	CN	10
RECA-EU	Renal Cell Cancer	EU/FR	95
SKCM-US	Skin Cutaneous melanoma	US	323
STAD-US	Gastric Adenocarcinoma	US	289
THCA-SA	Thyroid Cancer	SA	15
THCA-US	Head and Neck Thyroid Carcinoma	US	396

Cancer sequencing projects from ICGC data portal (https://dcc.icgc.org/) analyzed in this paper.

Supplementary Table 2

Cancer Type	A	B	C	r-value	p-value	STDERR	SM95
PanCancer	9.78E-01	2.17E-02	-3.53E-01	1.00	2.01E-65	6.86E-03	3.056
ESAD	1.00E+00	1.85E-06	-1.05E+00	1.00	4.42E-15	1.72E-02	0.006
MALY	1.00E+00	5.33E-06	-1.10E+00	1.00	1.60E-06	3.91E-02	0.023
RECA	1.00E+00	6.41E-05	-8.26E-01	0.99	3.73E-16	3.60E-02	0.030
LIRI	9.99E-01	6.25E-04	-5.95E-01	0.99	5.54E-38	2.43E-02	0.057
COAD	1.00E+00	4.02E-05	-1.01E+00	1.00	2.00E-49	5.61E-03	0.084
SKCM	9.99E-01	8.30E-04	-6.12E-01	1.00	5.48E-57	1.02E-02	0.111
PAEN	1.00E+00	4.54E-04	-7.97E-01	0.99	5.13E-03	6.95E-02	0.254
PACA	9.74E-01	2.57E-02	-1.93E-01	0.98	2.80E-36	2.88E-02	0.310
READ	1.00E+00	3.11E-04	-9.42E-01	1.00	1.11E-16	1.88E-02	0.428
PRAD	9.98E-01	1.82E-03	-7.54E-01	1.00	1.30E-25	1.25E-02	1.103
LINC	9.88E-01	1.25E-02	-4.08E-01	0.98	1.93E-36	2.91E-02	1.745
LGG	9.78E-01	2.25E-02	-3.29E-01	0.98	4.80E-33	3.53E-02	2.543
KIRP	9.98E-01	2.29E-03	-9.27E-01	1.00	1.34E-10	1.13E-02	3.250
GBM	9.97E-01	3.29E-03	-8.52E-01	1.00	1.24E-17	1.90E-02	3.597
ORCA	9.98E-01	1.85E-03	-1.02E+00	1.00	3.45E-02	5.40E-02	3.629
OV	9.82E-01	1.79E-02	-4.81E-01	1.00	3.26E-04	5.34E-02	5.766
ESCA	9.96E-01	4.04E-03	-9.63E-01	1.00	1.04E-03	1.64E-03	6.454
BLCA	9.88E-01	1.24E-02	-6.20E-01	0.99	4.15E-13	3.61E-02	6.894
BRCA	9.71E-01	2.90E-02	-4.23E-01	0.99	1.08E-46	1.70E-02	8.640
KIRC	9.92E-01	8.39E-03	-8.20E-01	1.00	3.24E-17	2.02E-02	8.903
LAML	9.64E-01	3.60E-02	-4.43E-01	1.00	4.09E-10	2.81E-02	13.037
BOCA	9.82E-01	1.81E-02	-6.78E-01	1.00	4.81E-22	1.29E-02	13.408
LUSC	9.43E-01	5.73E-02	-4.26E-01	0.96	3.91E-10	7.73E-02	21.640
STAD	8.88E-01	1.12E-01	-3.20E-01	0.96	1.68E-22	4.87E-02	30.172
THCA	3.47E-09	1.00E+00	-4.70E-02	0.81	1.57E-12	6.66E-02	33.592
CMDI	8.50E-01	1.50E-01	-4.37E-01	0.99	9.12E-47	1.70E-02	50.288
PBCA	2.99E-01	7.01E-01	-2.51E-01	0.95	5.29E-02	2.48E-01	74.924

Recurrence analysis of somatic mutations for 27 cancer types and PanCancer. Fitting of the points reported in Fig 3 of the main text. The curves describe the trend in the decrease of *Fraction of Donors* as a function of the *Fraction of Somatic Mutations* for each tumor type and for the PanCancer dataset. A, B and C are the parameters of the equation $Y=1/(A-B \bullet X^C)$. SM95 is the percentage of somatic mutations needed to recover 95% of the donors. This SM95 value is estimated using the previous equation.

Supplementary Table 3

Cancer Type	Exome Mutations (median)	Exonic Recurrent Somatic Mutations	Affected Genes	Affected Donors
BLCA	168.0	3,720	2,779	232 (99.6%)
BOCA	16.5	98	79	57 (86.4%)
BRCA	42.0	5,881	4,246	1,037 (96.8%)
CLLE	12.0	105	98	66 (60.6%)
CMDI	1.0	17	4	114 (88.4%)
COAD	135.0	15,753	8,620	216 (100.0%)
EOPC	55.0	39	38	11 (100.0%)
ESAD	454.0	1,830	1,508	95 (100.0%)
ESCA	80.5	486	430	81 (92.0%)
GACA	81.0	56	57	9 (100.0%)
GBM	74.0	2,329	1,775	268 (100.0%)
KIRC	64.0	2,160	1,597	400 (99.0%)
KIRP	84.0	1,066	868	156 (100.0%)
LAML	15.5	6,736	4,460	67 (89.3%)
LGG	45.0	1,930	1,481	278 (99.6%)
LIAD	26.5	223	218	29 (96.7%)
LICA	100.0	2,620	2,083	27 (93.1%)
LINC	168.5	3,538	2,950	244 (100.0%)
LIRI	267.5	1,459	1,288	206 (99.0%)
LUSC	285.0	3,508	2,643	288 (99.7%)
MALY	141.0	308	281	44 (100.0%)
NBL	2.0	10	8	11 (26.8%)
ORCA	79.5	380	344	50 (100.0%)
OV	154.0	872	737	178 (98.3%)
PACA	75.5	3,489	2,707	496 (98.4%)
PAEN	116.0	165	161	31 (88.6%)
PBCA	5.0	450	417	152 (61.3%)
PRAD	54.5	2,446	1,935	264 (100.0%)
READ	116.5	3,474	2,776	80 (100.0%)
RECA	158.0	506	404	103 (98.1%)
SKCM	409.0	18,654	7,644	323 (100.0%)
STAD	158.0	10,941	6,681	287 (99.3%)
THCA	16.0	9,524	4,834	373 (90.8%)
PanCancer	70.0	62,206	17,381	6,273 (95.3%)

Exome Mutations: median of the distribution of somatic mutations in exonic regions held by a donor. *Exonic Recurrent Somatic Mutations*: Total number of exonic mutation occurring in at least two donors. Affected Genes and Donor: Total number of genes and donors in affected by an *Exonic Recurrent Somatic Mutation*. The *Mutation Recurrence* has been calculated on the PanCancer dataset.
